# Combinatorial therapy with two pro-coagulants and one osmotic agent reduces the extent of the lesion in the acute phase of spinal cord injury in the rat

**DOI:** 10.1186/s40635-017-0164-z

**Published:** 2017-12-11

**Authors:** Mathieu Boutonnet, Elisabeth Laemmel, Eric Vicaut, Jacques Duranteau, Marc Soubeyrand

**Affiliations:** 1 Service de Réanimation, Hôpital d’Instruction des Armées Percy, Clamart, France; 2 INSERM U942, Equipe universitaire 3509 Paris VII-Paris XI-Paris XIII, Microcirculation, Bioénergétique, Inflammation et Insuffisance circulatoire aiguë, Paris Diderot-Paris VII, Paris, France; 3 Département d’Anesthésie Réanimation, Service de Réanimation, Hôpital de Bicêtre, Hôpitaux Universitaires Paris-Sud, Assistance Publique-Hôpitaux de Paris, Le Kremlin-Bicêtre, France; 4 Service de Chirurgie Orthopédique, Hôpital de Bicêtre, Hôpitaux Universitaires Paris-Sud, Assistance Publique-Hôpitaux de Paris, Le Kremlin-Bicêtre, France

**Keywords:** Spinal cord injuries, Combined modality therapy, Rats, Tranexamic acid, Fibrinogen, Saline solution hypertonic

## Abstract

**Background:**

Spinal cord injury (SCI) is a complex disease that leads to a motor, sensitive, and vegetative impairment. So far, single therapies are ineffective for treating SCI in humans and a multifactorial therapeutic approach may be required. The aim of this work was to assess the effect of a triple therapy (TT) associating two pro-coagulant therapies (tranexamic acid and fibrinogen) with an anti-edema therapy (hypertonic saline solution), on the extent of the lesion 24 h post-injury.

**Methods:**

The design of this study is a randomized controlled study. The setting of this study is an experimental study.

Male Wistar rats were assigned to receive saline solution for the control group or one of the treatment, or a combination of two treatments or the three treatments (triple therapy group (TT)). Animals were anesthetized and received a weight-drop SCI induced at the level of the 12th thoracic vertebra (Th12). They were treated by single therapies, double therapies, or TT started 5 min after the SCI.

**Results:**

The extent of the lesion was assessed 24 h after injury by spectrophotometry (quantification of parenchymal hemorrhage and blood-spinal cord barrier disruption) and by histology (quantification of spared neuronal tissue).

As compared with the control group, the TT significantly reduced parenchymal hemorrhage (*p* < 0.05) and improved the total amount of intact neural tissue, measured 24 h later (*p* = 0.003).

**Conclusions:**

Combinatorial therapy associating two pro-coagulants (tranexamic acid and fibrinogen) with an anti-edema therapy (hypertonic saline solution) reduces the extent of the lesion in the acute phase of spinal cord injury in the rat.

## Background

Traumatic spinal cord injury (TSCI) is a major cause of mortality and morbidity worldwide. TSCI leads to a motor, sensitive, and vegetative impairment [1] with a devastating impact on the patient and on the society [2]. Following the primary injury, the lesion progressively extends (i.e., secondary injury) notably as a consequence of parenchymal hemorrhage (PH) [3] and edema [4] with an increase of the intraspinal pressure associated with a decrease in spinal cord perfusion leading to ischemia. Given the somatotopic organization of the motor and sensitive pathways, early limitation of the lesion’s size is paramount to improve the functional prognosis of the patients [5]. So far, single therapies are ineffective for treating SCI in humans [6], probably because of the multi-factorial nature of secondary injury. To overcome these limits, combinatorial approaches may represent a promising alternative [7].

We made the hypothesis that combination of pro-coagulant therapies (tranexamic acid (TXA) and fibrinogen (Fg))—for limiting the extent of PH—with an anti-edema therapy (hypertonic saline solution (HTS)) may limit the spatial extent of the lesion. We designed the study with an early administration of the treatments such as it could be achieved in the prehospital setting. The aim of this work was to assess the effect of this triple therapy (TT) on the extent of the lesion 24 h post-injury.

## Methods

The animal bioethics board of the Lariboisière-Villemin School of Medicine approved this experimental study. The “Principles of laboratory animal care” (NIH publication No. 86-23, revised 1985) were followed. The animals were housed in individual cages in a room with a 12-h dark/light cycle and had free access to food and water.

### Surgical preparation

Male Wistar rats were anesthetized in an induction cage (Harvard Apparatus®) with isoflurane 5% and a 50/50 O_2_/N_2_O gas mixture. Rats were weighted and received a subcutaneous injection of buprenorphine 50 μg kg^−1^. Anesthesia was maintained with isoflurane 2% and the same gas mixture through a rostral mask connected to the anesthesia system. Jugular vein catheterization was performed in a supine position. Then rats were installed in a prone position on a heating blanket to maintain body temperature at 37 °C. The animals were fastened to the mask by the incisor teeth. The mask was connected to a custom-made surgical frame [8]. A cutaneous incision was performed from the 11th thoracic vertebra (Th11) to the first lumbar vertebra (L1). A thoracic laminectomy was performed at Th12 level in order to expose the spinal cord. At the end of the laminectomy, a 30 min stabilization period was started (isoflurane 1.5%; O_2_ 0.4 L min^−1^; N_2_O 0.4 L min^−1^).

### Spinal cord injury and treatment

TXA is a synthetic analog of lysine, which inhibits fibrinolysis by blocking lesion sites of lysine to plasminogen. Used in the first hours after a traumatic hemorrhagic shock, it allows a reduction of mortality [9].

Fg is a plasma glycoprotein of 340 kDa, made of two subunits. It is involved in both the primary and secondary hemostasis and is an hemostatic agent routinely used [10]. The two subunits of Fg are cleaved by thrombin in fibrinopeptids A and B, which polymerize to form the fibrin chains. In addition, the fibrin increases the firmness of the clot and the platelet aggregation by activating the glycoprotein GPIIb/IIIa [11].

Sodium chloride 7.5% (HTS) is a crystalloid fluid used for small volume resuscitation by creating an osmotic gradient pressure between the vascular system and the interstitium [12]. It is also used as an osmotic agent to reduce tissue edema in case of intracranial hypertension [13].

Animals were randomly assigned to one of the nine following groups.

In the Sham group, the animals were operated but did not receive TSCI. In all the other animals, TSCI was performed using a custom-made weight dropping impaction device [14] allowing the single drop (i.e., without bounce) of a 16-g weight on the spinal cord from a height of 5 cm.

The treatment started 5 min after TSCI and lasted 20 min allowing two perfusions of 2 mL kg^−1^ during 10 min. The total volume infused was equal to 4 mL kg^−1^. The doses used were 250 mg kg^−1^ for Fg, 100 mg kg^−1^ for TXA, and 2 mL kg^−1^ for HTS.

In the control group, the animals received a perfusion of normal saline solution (NSS) (4 mL kg^−1^ during 20 min), starting 5 min after TSCI.

In the Fg group, the animals received a first perfusion of Fg and a second perfusion of NSS.

In the TXA group, the animals received a first perfusion of TXA and a second perfusion of NSS.

In the HTS group, the animals received a first perfusion of NSS and a second perfusion of HTS.

In the Fg + TXA group, the animals received a first perfusion of Fg + TXA and a second perfusion of NSS.

In the Fg + HTS group, the animals received a first perfusion of Fg and a second perfusion of HTS.

In the TXA + HTS group, the animals received a first perfusion of TXA and a second perfusion of HTS.

In the TT group, the animals received a first perfusion of TXA + Fg and a second perfusion of HTS.

Muscles and incision were sutured, and rats were awoken and returned back to their individual cages. During the 24 following hours, the animals’ bladders were manually emptied twice and rats received two subcutaneous injections buprenorphine (50 μg kg^−1^).

### Preparation for spectrophotometric analysis

Rats were anesthetized 23 h and 30 min after the impact to provide an assessment at the 24th hour post-SCI. They were injected with a bolus of Evans blue dye (EB) 2% (1 mL/300 g) through the right femoral vein. A 30-min period was respected, and rats were exsanguinated by cardiac perfusion of NSS (250 mL at a rate of 50 mL min^−1^ to avoid any endothelial lesion). A 15-mm sample of spinal cord centered on the epicenter was harvested, weighted, and crushed to powder. The mixture was diluted in NSS (in a volume equal at nine times the weight of the sample), homogenized with a sonicator (Vibra Cell® 72,442; power 40%, pulse on 2.5 s/off 1 s, time 120 s).

### Spectrophotometric quantification of parenchymal hemorrhage

The homogenized solution was centrifuged at 14,000 rpm for a 30-min period. Drabkin method: The supernatant was collected and divided in aliquots (25 μL of supernatant diluted into 225 μL of Drabkin’s reagent, Sigma-Aldrich Co.®) into a 96-well glass plate. Drabkin’s reagent lyses red blood cells and reacts with all forms of hemoglobin except sulfhemoglobin, converting them to cyanmethemoglobin, a stable brownish-colored compound [15]. Colorimetric measurements were performed using a PerkinElmer® spectrophotometer (Victor™ X3 at the maximum of absorption for Drabkin’s reagent (560 nm)). The results were normalized to initial samples weight. The amount of extravasated blood—i.e., related to the size of the PH—was evaluated by calculating the hemoglobin concentrations on the basis of a standard curve of Drabkin’s reagent with known quantities of hemoglobin in uninjured exsanguinated spinal cord. The results were expressed in mass of hemoglobin per mass of spinal cord (μg mg^−1^).

### Spectrophotometric quantification of blood-spinal cord barrier disruption (BSCB)

The extent of BSCB disruption was evaluated by examining vascular permeability which is proportional to the total amount of extravascular EB [16]. A volume of 100 μL of the homogenized solution was diluted in 900 μL of dimethylformamid. This operation was repeated three times. The samples were centrifuged at 14,000 rpm for a 30-min period.

EB method: The supernatant was collected and divided in aliquots (150 μL) into a 96-well glass plate. The colorimetric measurements were performed using the same spectrophotometer at the absorption maximum for EB (620 nm). The results were normalized to initial sample weight, and EB concentrations were calculated on the basis of a standard curve of EB in saline solution. The results were expressed in mass of EB per mass of spinal cord (ng mg^−1^).

### Histologic examination

The rats were exsanguinated by cardiac perfusion of NSS (50 mL min^−1^) during 1 min followed by 4% paraformaldehyde during 4 min (to avoid any endothelial lesion). The laminectomy was extended, and a 20-mm-long fragment of spinal cord centered on the epicenter was harvested and post-fixed in 4% paraformaldehyde.

The fragments were placed in paraffin. Transverse sections of 4 μm thickness were cut every millimeter and stained with hematoxylin, phloxin, and saffron. The histological slides were scanned using CaloPix®. We evaluated the spared neural tissue using gray scale analysis. Spinal cord tissues were surrounded, and gray level analysis was performed using Image J® software. We assessed spared neural tissue on nine sections for each spinal cord fragment (the epicenter section and the four rostral and caudal ones).

### Statistical analysis

Data was analyzed using R® statistical software with the nparLD, nparcomp, and COIN packages (Version 3.1.3.).

All results are reported in median, (first-third quartiles), with *n* corresponding to the number of animals. Comparison of spectrophotometric results was made by exact permutation test between the control group and Sham group. Spectrophotometric results were compared by *k*-sample permutation test, and multiple comparisons were performed using the Dunnet correction. Amount of spared neural tissue was compared between the control group and TT group by exact permutation test. Distributions of gray level in the spinal cord were compared between the control group and TT group using rank based non-parametric methods for longitudinal data. Adjusted values of *p* lower than 0.05 were considered significant.

## Results

**Fig. 1:**
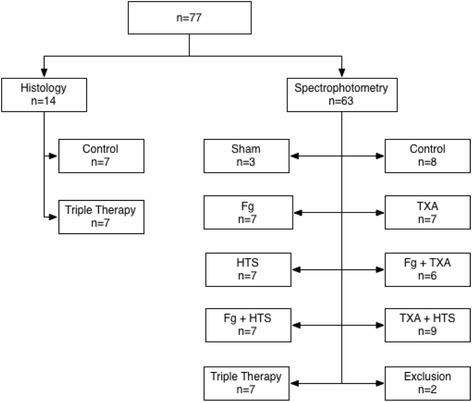
Flow chart diagram (two exclusions: one failed injection of Evans blue dye and one failed spinal cord injury)

A total of 77 rats were operated: 63 rats were included for spectrophotometric analyses and 14 for histologic assessments (Fig. 1).

### Spectrophotometric analysis

**Fig. 2:**
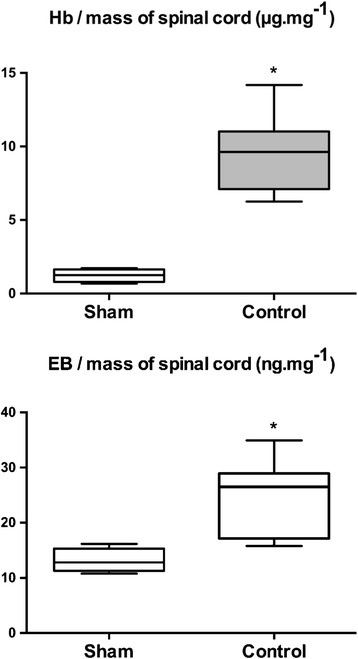
Parenchymal hemoglobin (upper) and Evans blue dye concentrations (lower) 24 h after spinal cord injury (mass of hemoglobin per mass of spinal cord (μg mg^−1^); mass of Evans blue dye per mass of spinal cord (ng mg^−1^), (*p* < 0.001 and *p* < 0.05)

Sham-operated animals had significantly lower rates of hemoglobin and EB than control animals, respectively, 1.1 μg mg^−1^ (0.9–1.4) vs 9.6 μg mg^−1^ (8.3–10.3) (*p <* 0.001) and 12.9 ng mg^−1^ (11.8–14.5) vs 26.5 ng mg^−1^ (21.2–27.8) (*p <* 0.05) (Fig. 2).

**Fig. 3:**
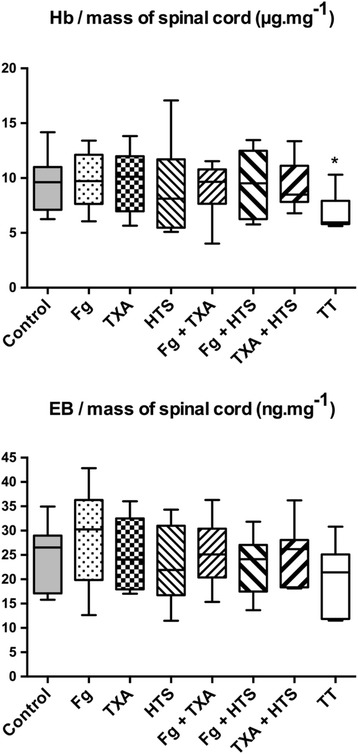
Parenchymal hemoglobin (upper) and Evans blue dye (lower) concentrations, 24 h after the spinal cord injury. Asterisk indicates statistically significant when compared with control (*p* < 0.05)

As compared with the control group, only the rats of the TT group had significantly lower rates of hemoglobin in the spinal cord (*p < 0.05*) (Fig. 3). We did not observe any differences among the treated groups regarding parenchymal EB quantities (Fig. 3).

### Histologic examination

**Fig. 4:**
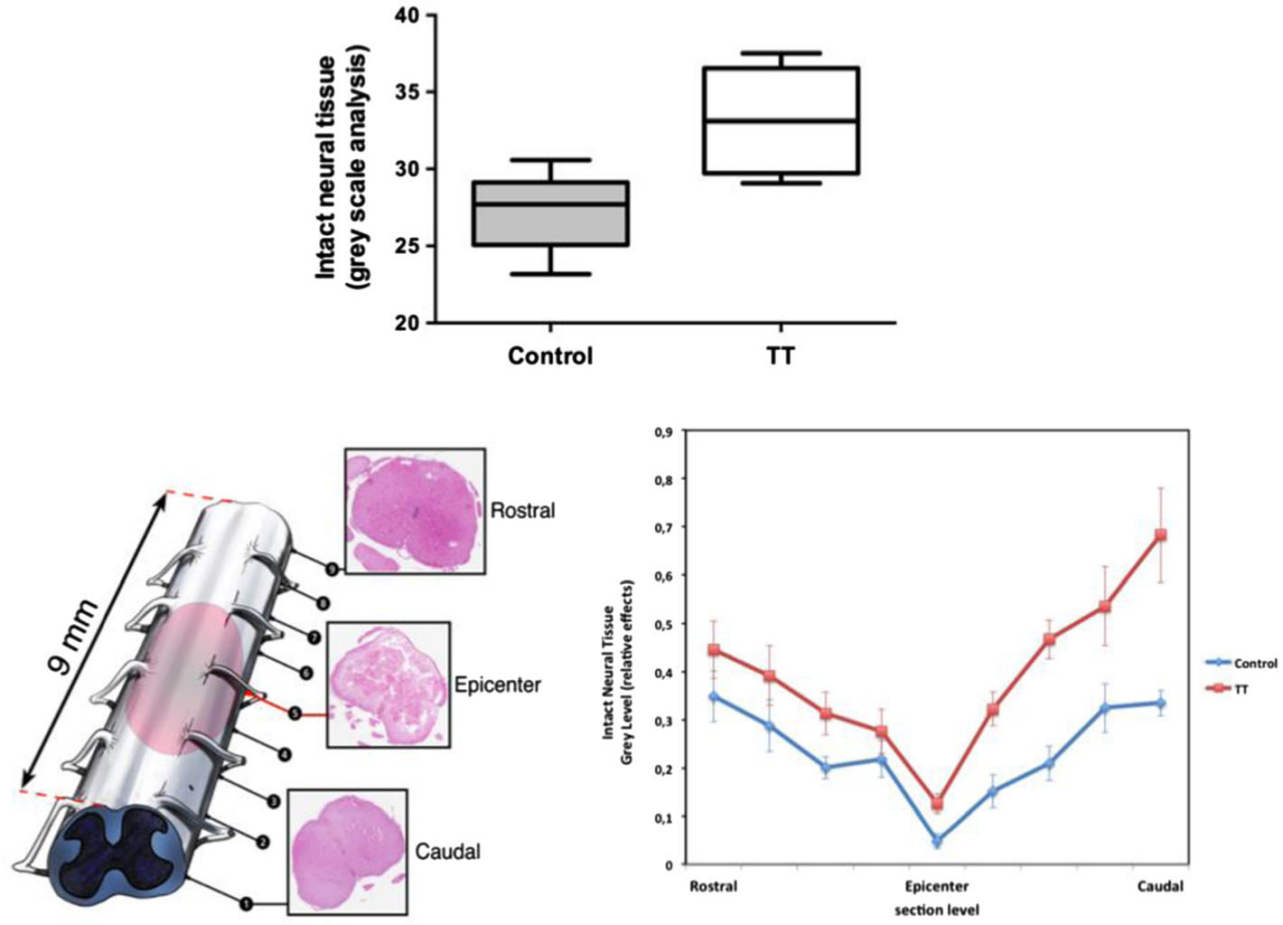
Total amount (upper) and distribution at each section level (lower) of intact neural tissue (gray scale analysis) in the control and triple therapy groups (*p* < 0.001 and *p* = 0.0001)

The amount of intact neural tissue, assessed by gray scale analysis, was significantly higher in the TT group than in the control group (*p* < 0.01). In addition, the distribution by section level of intact neural tissue enhanced a protective effect of the TT after SCI (*p =* 0.0001) (Fig. 4). We found that the TT limits the extension of the initial lesions and spares intact neural tissue.

## Discussion

As compared with the control group, the TT associating two pro-coagulant therapies (Fg and TXA) with an osmotic agent (HTS) resulted in a reduction of PH and greater preservation of neural tissue after spinal cord injury. In contrast, neither single therapies nor double therapies had an effect on the PH or EB extravasation.

To our best knowledge, this is the first time that a beneficial effect of pro-coagulant and osmotic therapies was demonstrated in the initial management of TSCI. The lack of effect of single and double therapies, while TT is effective, provides supplementary argument for using combinatorial approach in TSCI. Our finding emphasizes the fact that a unilateral approach of the TSCI pathophysiology is probably not sufficient. Our hypothesis was based on the fact that the complexity of the mechanisms involved requires a combinatorial therapeutic approach. Following a TSCI, a cascade of events contributes to create irreversible and extensive lesions. First, the primary injury due to the mechanical disruption of the structures of the spinal cord induces an immediate loss of neural tissue. Then, secondary injuries are triggered and worsen the lesion by destructing intact neighboring neural tissue [4]. Secondary injury starts with the hemorrhage into the spinal cord’s compartments (subdural, epidural, subarachnoid, and parenchymal spaces) [17]. Blood itself has an inherent toxicity for the central nervous system [18]. Following TSCI, ischemia appears in the surrounding area of the initial lesion by altered blood flow [19] and worsens as a result of local compression [20] by PH and edema due to the BSCBD [21] with a potential decrease in spinal cord perfusion due to an increase in intraspinal pressure. Ischemia may also result from vasospasm due to exposure to red blood cell components (oxyhemoglobin, endothelin) [22].

We are currently unable to explain how the therapies synergize to provide the beneficial effect observed with the TT. However, the lack of efficacy of single and double therapies, while TT is efficient, means that it is necessary not only to control the bleeding by strengthening the coagulation but also to reduce the associated edema. Acting on only one of these two pathways does not deliver any significant effect, which suggests that both mechanisms may potentiate. We propose as a hypothesis, that on the one hand, HTS, by reducing the parenchymal edema and the spinal cord swelling [13], could decrease the intra-spinal pressure thereby facilitating the venous outflow. This improvement in venous drainage would result in a decrease of the capillary pressure and would limit the rupture of their walls (i.e., breakdown of the blood spinal cord barrier) with a decrease in parenchymal hemorrhage. On the other hand, the combination of two pro-coagulant factors would speed up local hemostasis of ruptured capillaries thereby limiting the toxic effect of the PH and the spatial extent of the lesion.

The originality of our investigation was to test in combination three treatments, used daily in human practice and whose adverse effects are well known. In a larger prospect, it strongly suggests that other treatments, which previously failed to limit the extent of the lesion when used as single therapy, could actually become effective if combined with other drugs. The diversity of available drugs, as well as the way to combine them—in term of number of drugs and dosages—provides new prospects for neuroprotection in TSCI. This new approach, as opposed to the quest for new drugs—requiring by definition long processes of validation—leads to suppose faster translation to human since the drugs potentially used would be already mastered. Successful combinatorial strategies have already been performed in complex medical situations such as infectious disease [23]. Our promising findings require further investigations in rats and large animals before translation to humans, notably in regard to the doses and administration schema.

Indeed, we have chosen to study the pro-coagulant treatments using high doses, in order not to overshadow a modest effect. In the management of traumatic hemorrhagic shock in human, it is recommended that plasma fibrinogen concentrations should be greater than 1.5 to 2 g L^−1^ [24]. The doses of Fg we used were about eight times the doses used in human practice. However, such doses of human Fg concentrate have already been used in animals and notably in rats, with good pharmacodynamics properties and safety [11]. TXA is a fibrinolysis inhibitor and acts through another pathway on coagulation. Prophylactic TXA has been used with efficacy to reduce blood loss during and after spinal surgery in human [25]. TXA doses were about seven times the doses used in human practice. Such doses and higher (until 300 mg kg^−1^) have already been used in models of hemorrhage in rats with safety but variable efficacy [26]. A dose of 150 mg kg^−1^ of TXA has evidenced complete inhibition of clot lysis during more than 1 h [26].

HTS was used at a dose very close to the one usually required in human practice. It has been studied in hemorrhaged rats using higher doses than we used (HTS 7.5%, 4 mL kg^−1^) [27]. At the doses of 5 mL kg^−1^, HTS (7.5%) improved tissue oxygenation and perfusion and reduced systemic and pulmonary inflammatory response in rats treated for hemorrhagic shock [28]. Nevertheless, the application of our findings to humans will require the determination of the minimal effective dose in humans and of the optimal administration schema. The absence of adverse events will also have to be validated.

A limit of our study is the lack of neurobehavioral assessment, which precludes determining whether the TT can improve the neurological outcome. It is legitimate to ask whether the observed decrease in hematoma volume is sufficient to induce an effective improvement in sensory motor performance. However, it is well established in animals that the extent of the injury is the main determinant of the neurological functional recovery [5, 17]. In the cat, locomotion may recover in some case with the maintenance of a relatively small population of original axons (5–10%) [5]. Moreover, based on magnetic resonance imaging, it is admitted that extent of PH is correlated with poor functional outcome in human patients with SCI [29]. Therefore, we hypothesize that TT, by reducing the extent of the lesion, may lead to improvement in neurological outcome. Moreover, we choose to stop our study at 24 h and there is no proof that any beneficial effect would be long lasting or that any detrimental effect may appear at later time points. Thus, further studies should include neurobehavioral assessment at later time points. The use of crude histology and whole tissue measurement of hemorrhage prevents any mechanistic insight into the reported effect. Indeed, we did not report the volumetric effects of HTS, the infiltration of leukocytes, or the preservation of myelin tracts. These issues would deserve further investigations.

## Conclusions

Combinatorial therapy associating two pro-coagulants (TXA and Fg) with an anti-edema therapy (HTS) reduces the extent of the lesion in the acute phase of TSCI in the rat. These findings are a promising prerequisite for the use of combinatorial therapies in managing TSCI.

## Abbreviations

BSCB: Blood-spinal cord barrier disruption
EB: Evans blue dye
Fg: Fibrinogen
HTS: Hypertonic saline solution
L: Lumbar vertebra
NSS: Normal saline solution
PH: Parenchymal hemorrhage
Th: Thoracic vertebra
TSCI: Traumatic spinal cord injury
TT: Triple therapy
TXA: Tranexamic acid
